# Circulating Exosomal MicroRNA-1307-5p as a Predictor for Metastasis in Patients with Hepatocellular Carcinoma

**DOI:** 10.3390/cancers12123819

**Published:** 2020-12-18

**Authors:** Jung Woo Eun, Chul Won Seo, Geum Ok Baek, Moon Gyeong Yoon, Hye Ri Ahn, Ju A. Son, Suna Sung, Do Wan Kim, Soon Sun Kim, Hyo Jung Cho, Jae Youn Cheong

**Affiliations:** 1 Department of Gastroenterology, Ajou University School of Medicine, Suwon 16499, Korea; jetaimebin@gmail.com (J.W.E.); countpas@naver.com (C.W.S.); ptok99@hanmail.net (G.O.B.); ymk8028@hanmail.net (M.G.Y.); rhkwp37@naver.com (H.R.A.); gracia1429@ajou.ac.kr (J.A.S.); tjdtnsk1203@naver.com (S.S.); soonsunkim@aumc.ac.kr (S.S.K.); 2 Department of Biomedical Sciences, Ajou University Graduate School of Medicine, Suwon 16499, Korea; 3 Ajou Translational Omics Center, Ajou University Medical Center, Suwon 16499, Korea; kdowan@ajou.ac.kr

**Keywords:** hepatocellular carcinoma, metastasis, exosome, microRNA, bioinformatics analysis

## Abstract

**Simple Summary:**

Exosomal microRNAs (exo-miRs) significantly contribute to cancer metastasis. However, few studies have investigated the role of exosomes as metastasis mediators in hepatocellular carcinoma (HCC) despite recent advancements in liquid biopsy. We aimed to identify pro-metastatic circulating exo-miRs potentially predicting metastasis onset in HCC through comprehensive and systematic integrative analyses of plasma exo-miR sequencing data and publicly available RNA expression datasets, and accordingly propose a potential mechanism of action of pro-metastatic miRs, including promoting epithelial–mesenchymal transition (EMT). We found that circulating exo-miR-1307-5p is a predictive marker for metastasis in patients with HCC, and EMT promotion through SEC14L2 and ENG downregulation could be the potential downstream pathway of miR-1307-5p. We believe that our study makes a significant contribution to the literature because our findings provide novel insights into the role of circulating exo-miRs in the pathogenesis and progression of HCC and suggest that exo-miRs are a potential treatment target in HCC.

**Abstract:**

Exosomal microRNAs (exo-miRs) contribute to cancer metastasis. To identify pro-metastatic circulating exo-miRs in hepatocellular carcinoma (HCC), next-generation sequencing-based plasma exo-miR profiles of 14 patients with HCC (eight non-metastatic and six with metastasis within 1 year of follow-up) were analyzed. Sixty-one miRs were significantly overexpressed among patients with metastatic HCC. Candidate miRs were selected through integrative analyses of two different public expression datasets, GSE67140 and The Cancer Genome Atlas liver hepatocellular carcinoma (TCGA_LIHC). Integrative analyses revealed 3 of 61 miRs (miR-106b-5p, miR-1307-5p, and miR-340-5p) commonly overexpressed both in metastasis and vascular invasion groups, with prognostic implications. Validation was performed using stored blood samples of 150 patients with HCC. Validation analysis showed that circulating exo-miR-1307-5p was significantly overexpressed in the metastasis group (*p* = 0.04), as well as in the vascular invasion and tumor recurrence groups. Circulating exo-miR-1307-5p expression was significantly correlated with tumor stage progression (*p* < 0.0001). Downstream signaling pathways of miR-1307 were predicted using TargetScan and Ingenuity Pathway Analysis. On comprehensive bioinformatics analysis, the downstream pathway of miR-1307-5p, promoting epithelial–mesenchymal transition (EMT), showed SEC14L2 and ENG downregulation. Our results show that circulating exo-miR-1307-5p promotes metastasis and helps predict metastasis in HCC, and SEC14L2 and ENG are target tumor suppressor genes of miR-1307 that promote EMT.

## 1. Introduction

Hepatocellular carcinoma (HCC) is the sixth most common malignancy and the third leading cause of cancer-related mortality worldwide [1]. In the past several decades, the prognosis of HCC has significantly improved owing to advancements in diagnostic and treatment approaches for HCC [2]. However, the prognosis of patients with advanced-stage HCC remains poor with a median survival of 4–6 months [3]. Metastasis is a key determinant of the treatment strategy among patients with HCC because locoregional therapies are no longer effective to control extrahepatic metastasis [4]. Therefore, determination of the metastatic status during the initial staging process is essential for generating an appropriate treatment strategy directly associated with survival. In most cases, extrahepatic metastasis is detected through conventional imaging modalities including computed tomography and bone scintigraphy; however, they require considerable effort and are costly, and sometimes they cannot detect small metastatic lesions. The identification of metastasis driver molecules in blood before diagnosis through conventional imaging modalities would help classify patients in accordance with the stratified risk of metastasis, thus, potentially facilitating the implementation of precision medicine approaches.

Liquid biopsy is performed to detect tumor-derived genetic factors in body fluids including blood, urine, and saliva [5,6,7]. Liquid biopsy can target various classes of circulating tumor molecules including cell-free DNA, circulating tumor cells, and tumor cell-derived extracellular vesicles [8]. Exosomes (approximately 30–100 nm diameter) are extracellular vesicles delivering genetic factors from the donor cell to the recipient cell [9,10]. Exosomal content has been extensively assessed as major targets of liquid biopsy [11]. Among the exosomal genetic factors, microRNAs (miRs) received increasing attention because the loading of specific miRs into exosomes is suggested to result from active selection in accordance with the properties of donor cells [12,13]. Recent studies have shown that exosomes promote the generation of a metastatic niche by transferring functional molecules activating epithelial–mesenchymal transition (EMT) at different sites and promoting downstream signaling in recipient cells [14,15]. In particular, exosomal miRs (exo-miRs) significantly contribute to cancer metastasis. However, few studies have investigated the role of exosomes as metastasis mediators in HCC despite recent advancements in liquid biopsy.

In this study, we aimed to identify pro-metastatic circulating exo-miRs that potentially predict metastasis onset in HCC through comprehensive and systematic integrative analyses of plasma exo-miR sequencing data and publicly available RNA expression datasets. Accordingly, we propose a potential mechanism of action of pro-metastatic miRs, including promoting EMT.

## 2. Results

### 2.1. Confirmation of Isolated Circulating Exosomes and Identification of Overexpressed Exo-miRs in Metastatic HCC Patients

**Figure 1:**
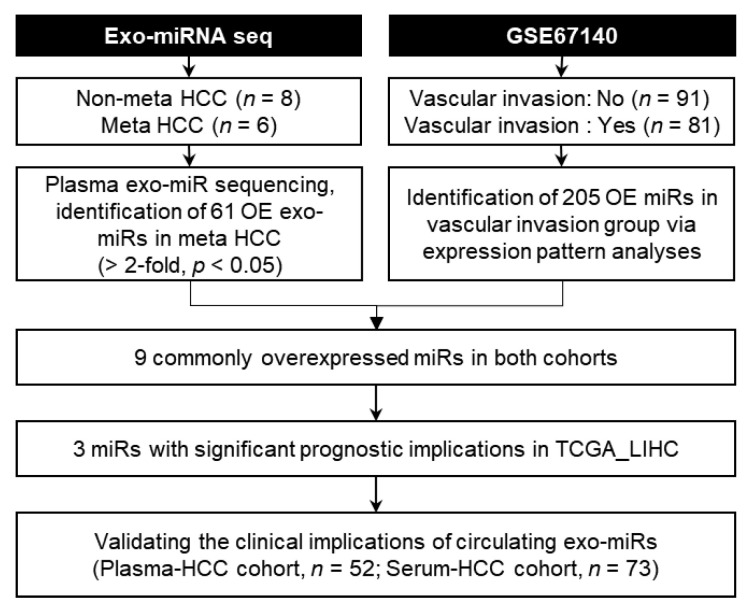
Schematic representation of the systematic integrative analyses performed herein to identify metastasis-stimulating circulating exosomal microRNAs. Exo-miRNA seq, exosomal microRNA sequencing; HCC, hepatocellular carcinoma; Meta, metastasis; exo-miR, exosomal microRNA; OE, overexpressed.

**Figure 2:**
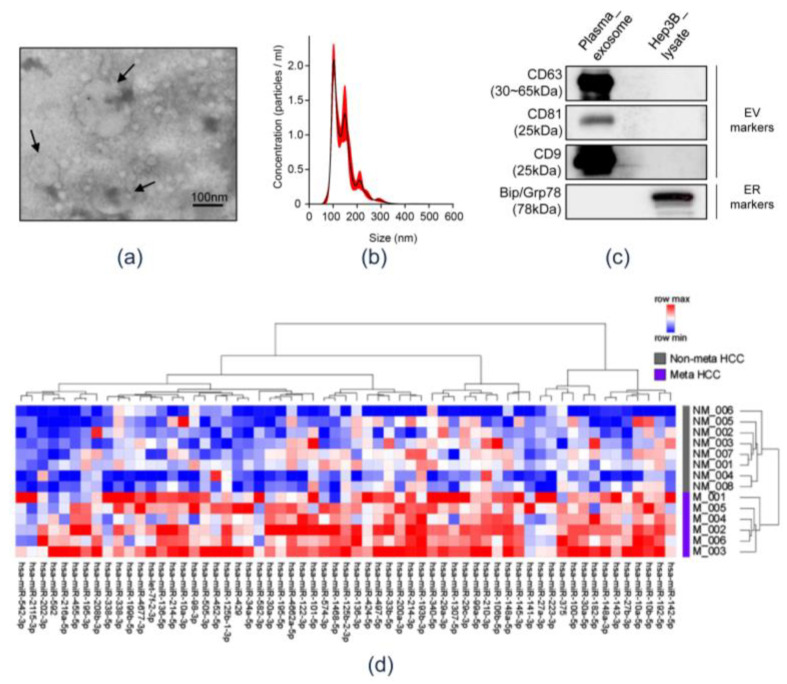
Confirmation of isolated circulating exosomes and identification of overexpressed exo-miRs in the metastasis group. (**a**) Transmission electron microscopic observation of separated circulating exosomes obtained from patients with hepatocellular carcinoma. Arrows indicate exosomes. Scale bar = 100 nm. (**b**) Nanoparticle tracking analysis (NTA) size distribution and concentration of exosomes. (**c**) Western blot analysis. Plasma exosomes were positive for exosome markers (CD63, CD81, and CD9) and negative for Grp78. Hep3B lysate was used as a negative control. (**d**) Heatmap of the differentially expressed 61 exo-miRs in accordance with the metastasis status. NM, non-metastasis; M, metastasis.

Figure 1 shows a schematic representation of systematic integrative analyses performed herein to identify circulating exo-miRs potentially promoting metastasis. First, circulating exosomes were isolated and their baseline characteristics were evaluated. Transmission electron microscopy (TEM) revealed that the samples contained spherical vesicles of 30–100 nm in diameter (Figure 2a). The concentration and size distribution of these vesicles were determined through Nanoparticle tracking analysis (NTA) (Figure 2b). NTA revealed that particles of 30–100 nm diameter were strongly enriched in the samples. The results of western blotting showed that the isolated vesicles were positive for exosomal markers (CD63, CD81, and CD9) and negative for an endoplasmic reticulum marker, Grp78 (Figure 2c and Figure S1). These results indicate that the circulating exosomes were well isolated, and exosomal RNA was appropriately extracted. Small RNA sequencing libraries were successfully generated from 14 of the 52 exosomal RNA preparation samples from the Plasma-HCC cohort. These 14 samples were used herein, of which, eight were obtained from the metastasis-free group and six from the metastasis group. Sequencing data of plasma exo-miRs were analyzed, and 61 exo-miRs predominantly overexpressed in the metastasis group were identified (>2-fold, *p* < 0.05). The heatmap reveals the overexpression of the 61 exo-miRs in the metastasis group (Figure 2d).

### 2.2. Integrative Analyses of Public Gene Expression Datasets to Select Potential Candidate Pro-Metastatic miRs

To further select pro-metastatic miRs, systematic integrative analyses were performed in two public gene expression datasets, GSE67140 and TCGA_LIHC [16]. The GSE67140 cohort comprised 91 patients with HCC with vascular invasion and 81 without vascular invasion. In the GSE67140 dataset, pattern analysis was performed in accordance with vascular invasion using CLICK algorithm [17].

**Figure 3:**
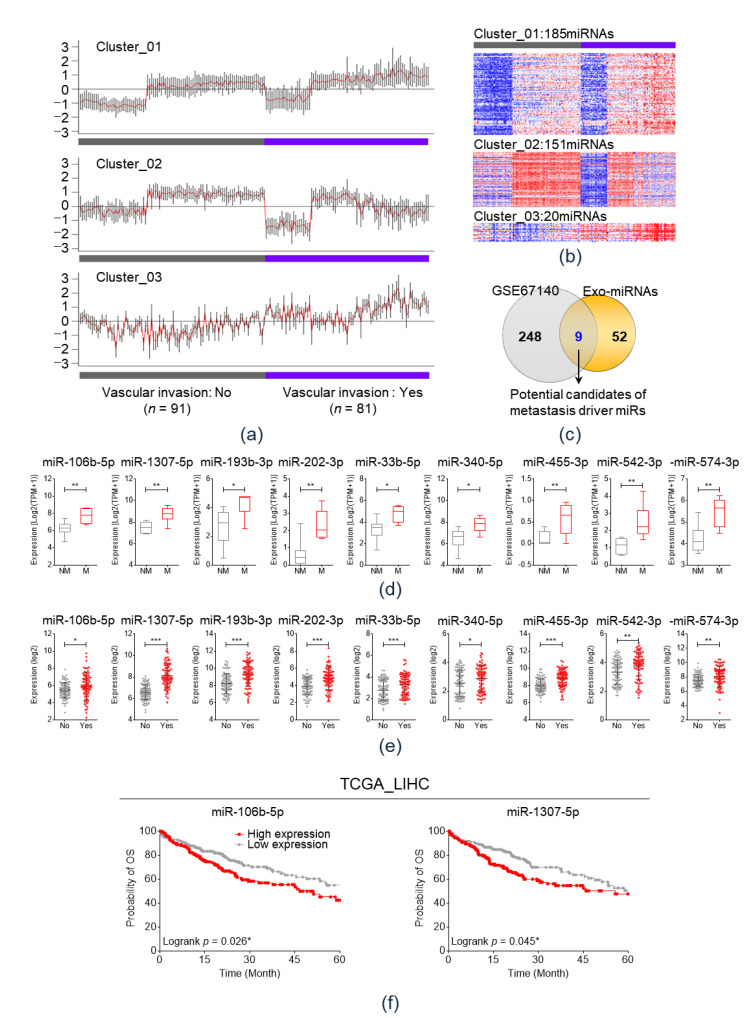
Integrative analyses of two different public RNA expression datasets (**a**) On Cluster analysis, miRs could be categorized into three clusters on the basis of their expression patterns according to vascular invasion status in GSE67140. (**b**) Heatmaps of miR expression in each cluster based on the vascular invasion status. (**c**) Venn diagram analysis to select potential candidate metastasis driver miRs. (**d**) Comparison of the expression of nine miRs between metastasis (M) and non-metastasis (NM) groups based on the expression of 14 plasma exosomal small RNA sequencing data. * *p* < 0.05; ** *p* < 0.01. (**e**) Comparison of the expression levels of nine miRs based on the vascular invasion status in the GSE67140 cohort. * *p* < 0.05; ** *p* < 0.01; *** *p* < 0.001. (**f**) Kaplan–Meier plot of overall survival based on the expression of miR-106b-5p and miR-1307-5p in TCGA_LIHC. * *p* < 0.05. NM, non-metastasis; Meta, metastasis; TCGA_LIHC, The Cancer Genomic Atlas Liver Hepatocellular Carcinoma.

As a result, miRs could be categorized into three clusters by the expression pattern according to vascular invasion status (Figure 3a). Figure 3b illustrates heatmaps of the miR expression in each cluster according to vascular invasion status. Among the three clusters, 185 miRs of cluster 1 and 20 miRs of cluster 3 showed significantly increased values in the vascular invasion group than in the non-vascular invasion group. Thus, the 205 miRs in cluster 1 or 3 were analyzed using a Venn diagram, which revealed that nine miRs—miR-106b-5p, miR-1307-5p, miR-193b-3p, miR-202-3p, miR-33b-5p, miR-340-5p, miR-455-3p, miR-542-3p, and miR-574-3p—were commonly overexpressed in both the metastasis group of the Plasma-HCC group and vascular invasion group of GSE67140 (Figure 3c).

The expression of the nine miRs in the 14 plasma exosomal small RNA sequencing (RNA-Seq) dataset was visualized in accordance with the metastasis status in Figure 3d. All nine miRs were significantly overexpressed in the metastasis group. Figure 3e shows the expression of the candidate miRs in accordance with their vascular invasion status in the GSE67140 cohort. All nine miRs were significantly overexpressed in the vascular invasion group. Using TCGA_LIHC datasets, the Overall survival (OS) was analyzed in accordance with the expression of the nine miRs (Figure 3f). As a result, overexpression of miR-106b-5p or miR-1307-5p was significantly associated with a poor OS; however, the expression levels of other miRs did not influence the OS.

### 2.3. Validation of the Clinical Implications of Candidate Exo-miRs in the Validation Cohort

**Figure 4:**
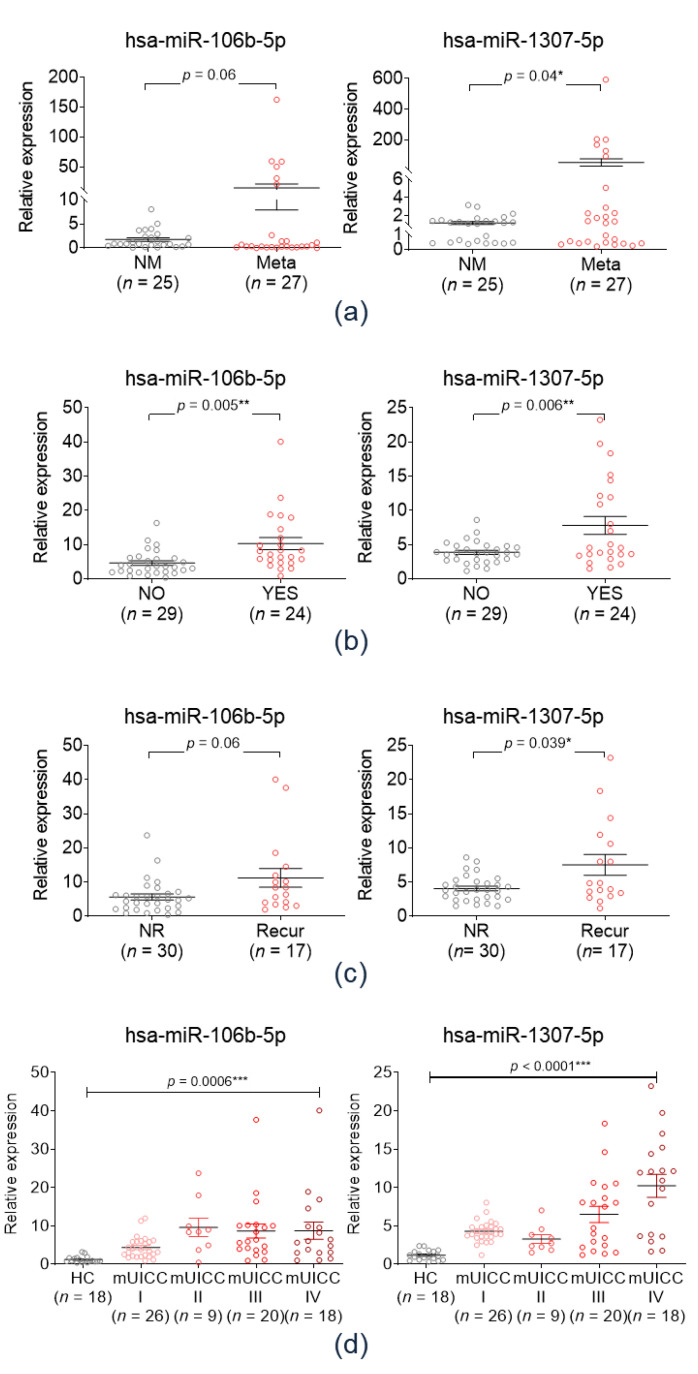
Validation of the clinical implications of candidate exo-miRs in the validation cohort. (**a**) Expression of two plasma exo-miRs in the metastasis-free group (NM, *n* = 27) and the metastasis group (Meta, *n* = 25). * *p* < 0.05. (**b**) Expression of the serum exo-miRs according to the vascular invasion status. * *p* < 0.05; ** *p* < 0.01. (**c**) Expression of serum exo-miRs according to the recurrence status. (**d**) Expression of the serum exo-miRs according to the modified UICC stage. *** *p* < 0.001. NM, non-metastasis; Meta, metastasis; UICC, Union for International Cancer Control.

Expression levels of plasma exo-miR-106b-5p and miR-1307-5p were determined in the Plasma-HCC cohort (*n* = 52) to validate the clinical implications of the selected miRs as metastasis predictors. Figure 4a shows the expression of the two plasma exo-miRs in the metastasis-free (*n* = 27) and metastasis groups (*n* = 25). Plasma miR-1307-5p expression levels were significantly higher in the metastasis group than in the non-metastasis group (*p* = 0.04), while that of exo-miR-106b-5p did not significantly differ between the two groups. 

Further validation analysis was performed to investigate the clinical implication of the selected circulating exo-miRs in the Serum-HCC cohort owing to its limited size and the lack of several clinical data regarding the Plasma-HCC cohort. Figure 4b shows the expression of exo-miR-106b-5p and miR-1307-5p in accordance with the vascular invasion status in the Serum-HCC cohort. Patients with vascular invasion had significantly higher serum exo-miR-106b-5p and miR-1307-5p expression levels. Figure 4c shows the expression of serum exo-miRs in accordance with the tumor recurrence status. Both of serum exo-miRs were significantly overexpressed in the tumor recurrence group. Figure 4d shows the expression of the three exo-miRs in accordance with the modified Union for International Cancer Control (mUICC) stages. Expression of the two serum exo-miRs were gradually upregulated according to tumor stage progression significantly.

Taken together, both of circulating exo-miR-1307-5p and exo-miR-106-5p were identified as potential biomarker for predicting vascular invasion, tumor recurrence, and advanced tumor stage in patients with HCC. However, in the aspect of metastasis, only exo-miR-1307-5p showed significant association with extrahepatic metastasis. Further, we tried to identify if circulating exo-miR-1307-5p could be used as a potential biomarker in the detection of HCC. Expression of serum exo-miR-1307-5p was compared between the HCC patients and normal healthy control (Figure S2). Serum exo-miR-1307-5p was markedly overexpressed in the HCC group compared to normal control (*p* < 0.0001). The AUC of exo-miR-1307-5p for detecting HCC was calculated as 0.958. 

### 2.4. In Silico Prediction of the Target Genes of miR-1307-5p

**Figure 5:**
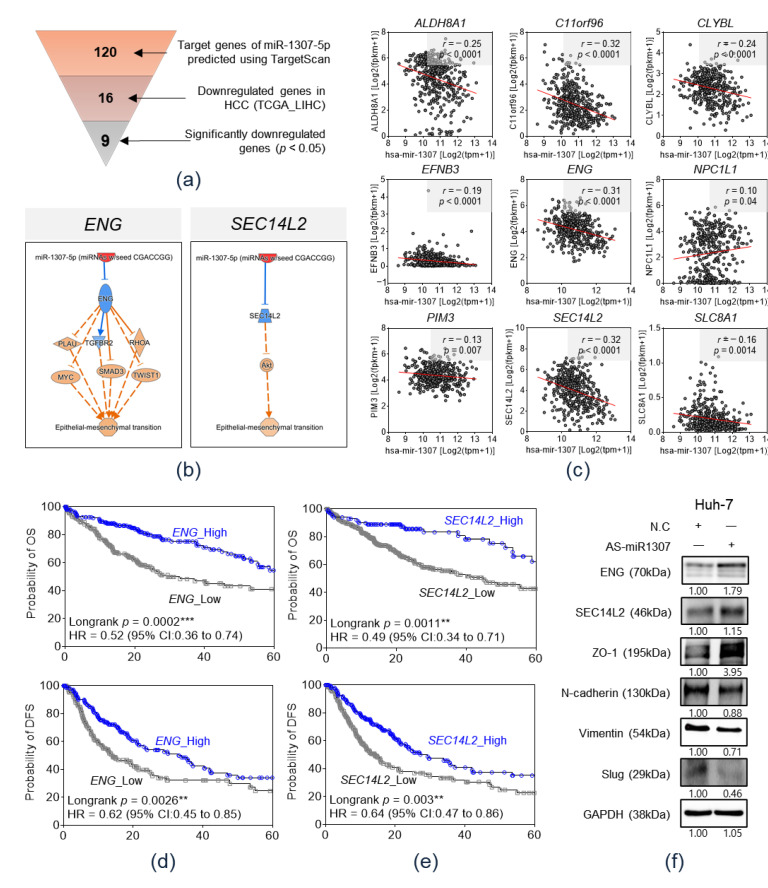
Prediction of target genes of miR-1307-5p in hepatocellular carcinoma through bioinformatics analysis. (**a**) Selection of potential target genes of miR-1307-5p via TargetScan 7.2 and expression data in TCGA_LIHC. (**b**) Pearson’s correlation analysis using the expression data in TCGA_LIHC to identify inversely correlated genes. (**c**) Pathway analysis with functional annotation of the epithelial–mesenchymal transition using the IPA software on miR-1307-5p and the target gene candidates, ENG and SEC14L2. (**d**) Kaplan–Meier plot of overall survival and disease-free survival based on ENG expression in TCGA_LIHC. ** *p* < 0.01; *** *p* < 0.001. (**e**) Kaplan–Meier plot of overall survival and disease-free survival based on SEC14L2 expression in TCGA_LIHC. ** *p* < 0.01. (**f**) Western blot analysis of ENG, SEC14L2, and EMT markers after miR-1307-5p inhibition by AS-miR-1307-5p in human HCC Huh-7 cells.

We attempted to identify the downstream target genes of miR-1307-5p that promote extrahepatic metastasis. Target gene prediction using TargetScan 7.2 identified 120 candidates as miR-1307-5p target genes (Figure 5a). Thereafter, the expression levels of 120 genes were evaluated in the TCGA_LIHC cohort. As miRs negatively regulate their target genes, we attempted to identify genes downregulated in the HCC tissues. In the TCGA_LIHC cohort, 16 of 120 genes were downregulated in HCC tissue rather than in adjacent non-tumor tissue, and 9 of 16 genes, namely, *ALDH8A1*, *C11orf96*, *CLYBL*, *EFNB3*, *ENG*, *NPC1L1*, *PIM3*, *SEC14L2*, and *SLC8A1*, displayed a significant difference (*p* < 0.05) (Figure 5a and Figure S3).

To identify genes inversely associated with miR-1307-5p, Pearson’s correlation analysis was performed using the expression data in the TCGA_LIHC database. The expression levels of five of nine genes, namely, *ALDH8A1*, *C11orf96*, *CLYBL*, *ENG*, and *SEC14L2*, displayed a significant inverse correlation with miR-1307-5p expression (*r* ≤ −0.3 and *p* < 0.05) (Figure 5b). We performed pathway analysis with functional annotation of the EMT, using the Ingenuity Pathway Analysis (IPA) software on miR-1307-5p and the five target candidate genes (Figure 5c and Figure S4). Consequently, miR-1307-5p/SEC14L2/Akt and miR-1307-5p/ENG signaling pathways were associated with the EMT. Survival analyses based on the expression of *ENG* and *SEC14L2* was performed using expression data from the TCGA_LIHC database. Figure 5d,e display Kaplan–Meier plots of OS and disease-free survival (DFS) based on the expression of ENG and SEC14L2, respectively. Compared to the high expression group, the low ENG expression group had a significantly poor OS (*p* = 0.0002) and poor DFS survival (*p* = 0.0026). Furthermore, compared to the high expression group, the low *SEC14L2* group had a poor OS (*p* = 0.011) and DFS (*p* = 0.003). 

To validate the downstream pathway of miR-1307-5p proposed by TargetScan and IPA, we treated antisense (AS)-miR-1307-5p, an inhibitor of miR-1307-5p, to immortalized HCC cell line- Huh-7 cell. Then, protein expression level of ENG and SEC14L2, which were proposed target molecules of miR-1307-5p, and expression of EMT markers (ZO-1, N-cadherin, vimentin, and slug) were evaluated by western blotting in Huh-7 (Figure 5f). As a result, expression level of ENG and SEC14L2 was increased by inhibition of miR-1307-5p, and the expressions of the EMT markers were altered as a direction of EMT promotion after treatment of AS-miR-1307-5p. Taken together, we could confirm the downstream pathway of miR-1307-5p in HCC, which down-regulates ENG/SEC14L2 and promotes EMT process. 

## 3. Discussion

Increasing evidence indicates that exosomes deliver pro-metastatic molecules to recipient cells, resulting in a pre-metastatic niche [14,15]. This study was based on the assumption that the expression of specific exo-miRs potentially increased during systemic circulation prior to extrahepatic metastasis, thus, promoting metastasis in patients with HCC.

To confirm this assumption, next generation sequencing-based circulating exo-miR profiles were analyzed, and differentially expressed circulating exo-miRs were identified between the metastasis-free group and the metastasis group during the follow-up period. Among the 61 predominantly overexpressed exo-miRs in the metastasis group, candidate miRs were further selected through systematic integrative analyses of publicly available RNA expression datasets. Consequently, exo-miR-1307-5p was identified as potential candidate pro-metastatic molecule. In the validation study, circulating exo-miR-1307-5p was significantly overexpressed in the metastasis group. Furthermore, SEC14L2 and ENG downregulation and the promotion of the EMT were considered potential downstream pathways of miR-1307-5p upon comprehensive bioinformatics analyses. We validated it by demonstrating up-regulation of ENG/SEC14L2 and EMT marker expression alteration after AS-miR-1307-5p treatment.

Exosomes contain unique cargo from donor cells, and exosomal cargo is considered a promising cancer biomarker. Several recent studies have shown circulating exo-miRs as potential diagnostic biomarkers for early-stage HCC [18,19]. Moreover, aberrantly regulated exo-miRs can promote HCC progression and metastasis by altering the genetic network [20]. An in vitro study by Lin et al., exosome-mediated miR delivery was shown to promote the EMT and metastasis in HCC [21]. However, few studies have investigated circulating exo-miR profiles as metastasis predictors or promoters in HCC. Herein, circulating exo-miR-1307-5p was considered a potential candidate metastasis predictor and metastasis driver in patients with HCC. Although miR-1307 is known as an onco-miR in diverse cancers as well as HCC, the clinical implication of the circulating exo-miR-1307-5p as cancer biomarker was only evaluated in ovarian cancer [22,23,24,25]. We identified exo-miR-1307-5p as a potential candidate metastasis driver and predictor in HCC. To our knowledge, this is the first study to identify circulating exo-miR-1307-5p as a novel metastasis promoter and predictor in patients with HCC.

Vascular invasion is considered as a pathognomonic hallmark of HCC invasiveness and poor prognosis [26]. Furthermore, it has been reported as a principal predictive marker for tumor recurrence and extrahepatic metastasis in HCC [27]. Herein, circulating exo-miR-1307-5p was significantly overexpressed in patients with vascular invasion as well as metastasis and tumor recurrence. Considering that vascular invasion is closely associated with subsequent extrahepatic metastasis and tumor recurrence in patients with HCC, exo-miR-1307-5p may potentially serve as a prognostic biomarker in patients with HCC.

Epithelial cells lose their epithelial phenotype and display a mesenchymal phenotype during the EMT [28]. EMT markedly promotes tumor invasiveness and metastasis by obliterating cell-cell adhesion [29]. Herein, we proposed SEC14L2/Akt and ENG-related signaling pathways as downstream pathways of miR-1307-5p for promoting the EMT in patients with HCC. Pathway analysis using IPA revealed that SEC14L2 is downregulated by miR-1307, in turn activating the Akt pathway, thus, promoting the EMT. SEC14L2 is a potent tumor suppressor gene in various malignancies [30]. Li et al. reported that SEC14L2, a novel master regulator gene, exerts an anti-proliferative effect in HCC cells and strongly suppresses tumor growth in a mouse model [31]. ENG (CD105), a transmembrane glycoprotein, is a transforming growth factor-β co-receptor [32]. ENG is involved in angiogenesis in solid tumors including HCC [33]. Several studies have shown that ENG downregulation in HCC tissue and its serum levels potentially serve as a poor prognostic marker in patients with HCC [32,34,35]. However, the mechanisms of action of ENG in HCC progression remain unclear. The present study demonstrated that miR-1307-5p down-regulates ENG and that downregulation of ENG is associated with the EMT promotion. 

This study has two limitations. First, the patient cohort sizes were small. Herein, the metastasis group included patients with extrahepatic metastasis after initial blood sampling. As patients with HCC with available blood samples prior to the occurrence of metastasis were rare, we could not enroll enough patients to obtain a high statistical power. Furthermore, owing to the shortage of blood samples from patients with metastatic HCC, we assume that the present results provide valuable and potentially useful information regarding pro-metastatic exo-miRs. Further validation studies with larger cohorts are needed to verify the present results. Second, we could not determine the mechanism underlying the promotion of extrahepatic metastasis by exo-miR-1307-5p. To overcome this limitation, we implemented an in silico analysis strategy. Downstream pathways of miR-1307-5p promoting the EMT were predicted through in silico analysis. Hence, further studies are required to confirm the underlying signaling pathway.

## 4. Materials and Methods

### 4.1. Patients and Sample Collection

To identify candidate circulating exo-miRs with pro-metastatic potential, the medical records of patients with HCC with available plasma samples during diagnosis were reviewed and patients were included in accordance with the inclusion and exclusion criteria. The inclusion criteria were as follows: (1) patients newly diagnosed with HCC in accordance with the American Association for the Study of Liver Diseases, criteria [36]; (2) patients without extrahepatic metastasis during diagnosis; (3) patients treated with local or systemic therapy in accordance with the tumor burden or location [37]; (4) Child-Pugh class A or B; and (5) the availability of follow-up imaging data for evaluating the tumor burden and metastasis status every 3 months for >1 year. Patients lost to follow-up 1 year before metastasis onset were excluded. Extrahepatic metastasis occurred in 25 of 52 patients meeting the inclusion criteria (metastasis group), and the remaining 27 patients were metastasis-free during follow-up evaluation (metastasis-free group). This cohort was called the Plasma-HCC cohort.

Owing to the low strength and lack of several clinical data in the Plasma-HCC cohort, patients with HCC with available pre-treatment serum samples during diagnosis were included to validate the clinical implications of selected circulating exo-miRs. This validation cohort was called the Serum-HCC cohort, comprising 91 serum samples from 73 patients with HCC and 28 healthy controls. A healthy control was defined as an individual without any medical history, who visited the Ajou Health Promotion Center for a regular health check-up. Data on the vascular invasion status, metastasis status, and tumor stage based on the modified Union for International Cancer Control (mUICC) staging system were obtained. The baseline characteristics of patients in the Plasma-HCC and Serum-HCC cohorts are elucidated in Table 1.

The study protocol was approved by the Institutional Review Board of the Ajou University Hospital, Suwon, South Korea (AJRIB-BMR-OBS-16-344). Anonymous blood samples and clinical data were provided by the Ajou Human Bio-Resource Bank. Informed consent was waived.

### 4.2. Analysis of Gene Expression Omnibus (GEO) Database and TCGA_LIHC

To estimate miR expression levels in HCC, public genomic data were obtained from TCGA_LIHC (https://cancergenome.nih.gov) and the GEO database (GSE67140) [16] of the National Center for Biotechnology Information.

### 4.3. Cell Culture and AS-miR-1307-5p Transfection

Hep3B and Huh-7 cells (ATCC, Manassas, VA, USA) were cultured in EMEM or DMEM mudium (GenDEPOT, Barker, TX, USA) containing 10% FBS (Invitrogen, Waltham, Massachusetts, USA) and 100 units/mL penicillin-streptomycin (GenDEPOT), at 37 °C in a humidified incubator with 5% CO_2_. Antisense inhibitor miR-1307-5p or scrambled control antisense inhibitor (Bioneer, Daejeon, Korea) was transfected into Huh7 cells using Lipofectamine 2000 (Invitrogen) according to the manufacturer’s protocol.

### 4.4. Blood Exosome Isolation and Total Exosomal RNA Extraction

Blood samples were obtained from the Biobank of Ajou University Hospital, a member of the Korea Biobank Network. Five milliliters of blood were collected from each individual directly into EDTA-containing tubes (for plasma) or serum-separating tubes (for serum) and centrifuged at 2000× *g* for 5 min at 4 °C and the resultant plasma or sera were aliquoted into 1.5 mL tubes and stored at −80 °C until use. To isolate exosomes from blood, the ExoQuick reagent-cat# EXOQ5A-1 (System Biosciences, Mountain View, CA, USA) was used for serum exosome isolation and the ExoQuick Plasma Prep and Exosome Precipitation Kit (Cat# EXOQ5TMA-1) was used for plasma exosome isolation. Briefly, plasma samples were ultra-centrifuged for 15 min (13,000 rpm) to remove partial cells and their debris. Then, to remove fibrinogen and fibrin in plasma, 5uL SBI Thrombin Reagent was added to the supernatant to convert fibrinogen into fibrin and centrifuged at 10,000× *g* for 5 min to make fibrin pellet. Then, the supernatant was transferred to a new microfuge tube and the fibrin pellet was discarded. Exosomes were isolated from the supernatant using the ExoQuick Exosome Precipitation Solution in accordance with the manufacturer’s protocol. Finally, the exosome pellet was re-suspended in 100 uL of PBS and stored at −80 °C for subsequent extraction of RNAs and proteins. RNA from blood-derived exosomes was extracted using the SeraMir Exosome RNA Amplification kit (System Biosciences). Thereafter, the total RNA concentration and purity were assessed using a NanoDrop 2000 spectrophotometer (Thermo Fisher Scientific, Waltham, MA, USA).

### 4.5. Small RNA Sequencing

Small RNA libraries were constructed from total RNA using the Illumina HiSeq 2000 system (Illumina Inc, San Diego, CA, USA). After small-RNA sequencing, reads were trimmed by cutadapt program for removing adapter and low-quality sequences, 18~26 bp in length considering the length of mature miRNA. Then, the trimmed reads were collapsed to remove duplicates and estimate abundance for the same sequence, and annotated using blast with miRBase. For comparison between samples, count of each sample was normalized in units of Transcripts Per Million (TPM).

### 4.6. TEM

TEM was performed to confirm the presence and sizes of exosomes. Samples were fixed in 2% glutaraldehyde and 4% paraformaldehyde for 2 h at room temperature and embedded responded with 0.13% methylcellulose and 0.4% uranyl acetate. Exosomes were then observed using a Hitachi H-7600 TEM (Hitachi High-Tech, Tokyo, Japan).

### 4.7. NTA

NTA was used to measure the size distribution and concentration of exosomes on the basis of light scattering and Brownian motion. NanoSight NS300 (Malvern Panalytical Ltd., Malvern, UK) equipped with a 405 nm laser with a frame rate of 30 frames/s was used for recording particle movement, and the data were evaluated using the NTA software (version 3.0, Malvern Panalytical Ltd.).

### 4.8. Western Blotting

Proteins were extracted from exosome and cell lysates, using radio immunoprecipitation (RIPA) buffer containing Halt Protease Inhibitor Cocktail (Thermo Fisher Scientific). Total proteins were separated by sodium dodecyl sulfate polyacrylamide gel electrophoresis and transferred to a polyvinylidene difluoride membrane (Merck Millipore, Burlington, Massahusetts, USA). The membranes were blocked with 5% non-fat milk in Tris-buffer saline and 0.1% Tween-20 and probed with the following primary antibodies: mouse anti-CD63 (1:1000, Abcam, Cambridge, MA, USA), rabbit anti-CD9 (1:2000, Abcam), mouse anti-CD81 (1:250, Invitrogen), and mouse anti-Bip/Grp78 (1:1000, BD Biosciences, San Jose, CA, USA), rabbit anti-ENG (1:1000, Abcam), rabbit anti-SEC14L2 (1:1000, Abcam), mouse anti-ZO-1 (1:1000, Thermo Fisher Scientific), mouse anti-N-cadherin (1:2000, BD bioscience), rabbit anti-Vimentin (1:5000, GeneTex, Alton, CA, USA), rabbit anti-Slug (1:1000, Cell Signaling Technology, Danvers, MA, USA), and mouse anti-GAPDH (1:1000, Santa Cruz Biotechnology, Santa Cruz, CA, USA). Chemiluminescence signals were detected using Clarity™ Western ECL Substrate and ChemiDoc (both from Bio-Rad Laboratories, Hercules, CA, USA). 

### 4.9. Quantitative Reverse-Transcription Polymerase Chain Reaction (qRT-PCR) Analysis

Circulating exo-miR expression levels were quantified using qRT-PCR. Each miR sequence was obtained from the miRBase database. [38] Primer sequences used herein are listed in Table S1. cDNA was synthesized from exosomal RNA using the miScript RT II kit (Qiagen, Hilden, Germany) in accordance with the manufacturer’s instructions, amplified using the Amfisure qGreen qPCR Master Mix (GenDEPOT), and monitored in real time using CFX Connect™ Real-Time PCR Detection System (Bio-rad Laboratories). The cycling conditions were as follows: 2 min at 95 °C, 40 cycles of 15 s at 95 °C, 34 s at 58 °C or 60 °C, and 30 s at 72 °C. Individual miR expression levels were determined from triplicate reactions and normalized with that of hsa-miR-1228-3p. The relative standard curve method (2−ΔΔCT) was used to determine relative expression levels.

### 4.10. Prediction of miR Targets

miR-1307-5p targets were predicted in silico using TargetScan 7.2 [39].

### 4.11. IPA

Signaling pathways downstream of miR-1307-5p and its target genes were subjected to functional annotation of EMT via IPA (Qiagen Inc., Redwood City, CA, USA) [40].

### 4.12. Statistical Analysis

All experiments were performed at least three times and all samples were analyzed in triplicate. Between-group differences were analyzed using a paired *t*-test or unpaired Welch’s *t*-test with GraphPad prism version 5.0 software (GraphPad Software Inc, San Diego, CA, USA). OS and DFS were plotted using the Kaplan–Meier method, and the significant differences were analyzed using the log-rank test. Differences were considered statistically significant when *p* was < 0.05.

## 5. Conclusions

In conclusion, this study shows that circulating exo-miR-1307-5p is a novel metastasis promoter and predictive marker for metastasis in patients with HCC, through systematic integrative analyses. EMT promotion through SEC14L2 and ENG downregulation could be the potential downstream pathway of miR-1307-5p, as revealed through comprehensive bioinformatics analyses.

## Figures and Tables

**Table 1:** Baseline characteristics of the Plasma-HCC cohort and the Serum-HCC cohort.

Variables	Plasma-HCC Cohort (*n* = 52)	Serum-HCC Cohort (*n* = 91)	
	HCC (*n* = 52)	Healthy Control (*n* = 28)	HCC (*n* = 73)
Age (years), mean ± SD	54.22 ± 9.98	34.96 ± 8.18	54.62 ± 9.06
Male sex, *n* (%)	47 (85.5)	3 (10.7)	57 (78.1)
AST, IU/mL	72.73 ± 77.36	16.82 ± 4.06	73.81 ± 92.28
ALT, IU/mL	46.11 ± 32.85	14.14 ± 7.77	49.48 ± 63.53
Platelet, x109/L	161.59 ± 82.04	314± 63.33	169.85 ± 85.41
AFP (ng/mL), mean ± SD	8000.26 ± 17,325.08	1.65 ± 0.58	4394.77 ± 14,600.73
Etiology, *n* HBV/HCV/alcohol/others	–		65/4/3/1
Albumin (g/L), mean ± SD	4.07 ± 0.60		4.23 ± 0.55
Bilirubin (mg/dL), mean ± SD	1.22 ± 1.81		1.49 ± 3.91
INR, mean ± SD	1.16 ± 0.14		1.18 ± 0.19
Modified UICC stage, *n* (%)	–		26 (36)/9 (12)/20 (27)/11 (15.4)/7 (9.6)
I/II/III/IVa/IVb			
Metastasis, *n* (%)	25 (48.1)/27 (51.9)		7 (9.6)/66 (90.4)
Yes/No			
Vascular invasion, *n* (%)	–		29 (54.7)/24 (45.3)
Yes/No			

HCC, hepatocellular carcinoma; AST, aspartate transaminase; ALT, alanine transaminase; AFP, alpha-feto protein; HBV, hepatitis B virus; HCV, hepatitis C virus; INR, international normalized ratio; UICC, Union for International Cancer Control; ⎻ Dashes denote lack of reliable data.

## Supplementary Materials

The following are available online at https://www.mdpi.com/2072-6694/12/12/3819/s1, Figure S1: The whole blot (uncropped blots) showing all the bands with all molecular weight markers, Figure S2: Serum exo-miR-1307-5p expression and ROC curves in normal healthy controls and the patients with HCC, Figure S3: Expression of the nine target gene candidates of miR-1307-5p in The Cancer Genomic Atlas Liver Hepatocellular Carcinoma (TCGA_LIHC), Figure S4: Pathway analysis with a functional annotation of the epithelial–mesenchymal transition (EMT) using the Ingenuity Pathway Analysis (IPA) software on miR-1307-5p and the target gene candidates, *ALDH8A1*, *C11orf96*, and *CLYBL*, Table S1: miRNA sequence used for qRT-PCR.
