# Supplementary material for: Circulating Exosomal MicroRNA-1307-5p as a Predictor for Metastasis in Patients with Hepatocellular Carcinoma
Source: Cancers (Basel). 2020 Dec 18;12(12):3819. doi: 10.3390/cancers12123819 (PMC7766543; doi:10.3390/cancers12123819)
Supplement: Supplementary file 1 [file cancers-12-03819-s001.pdf]

Supplementary Materials

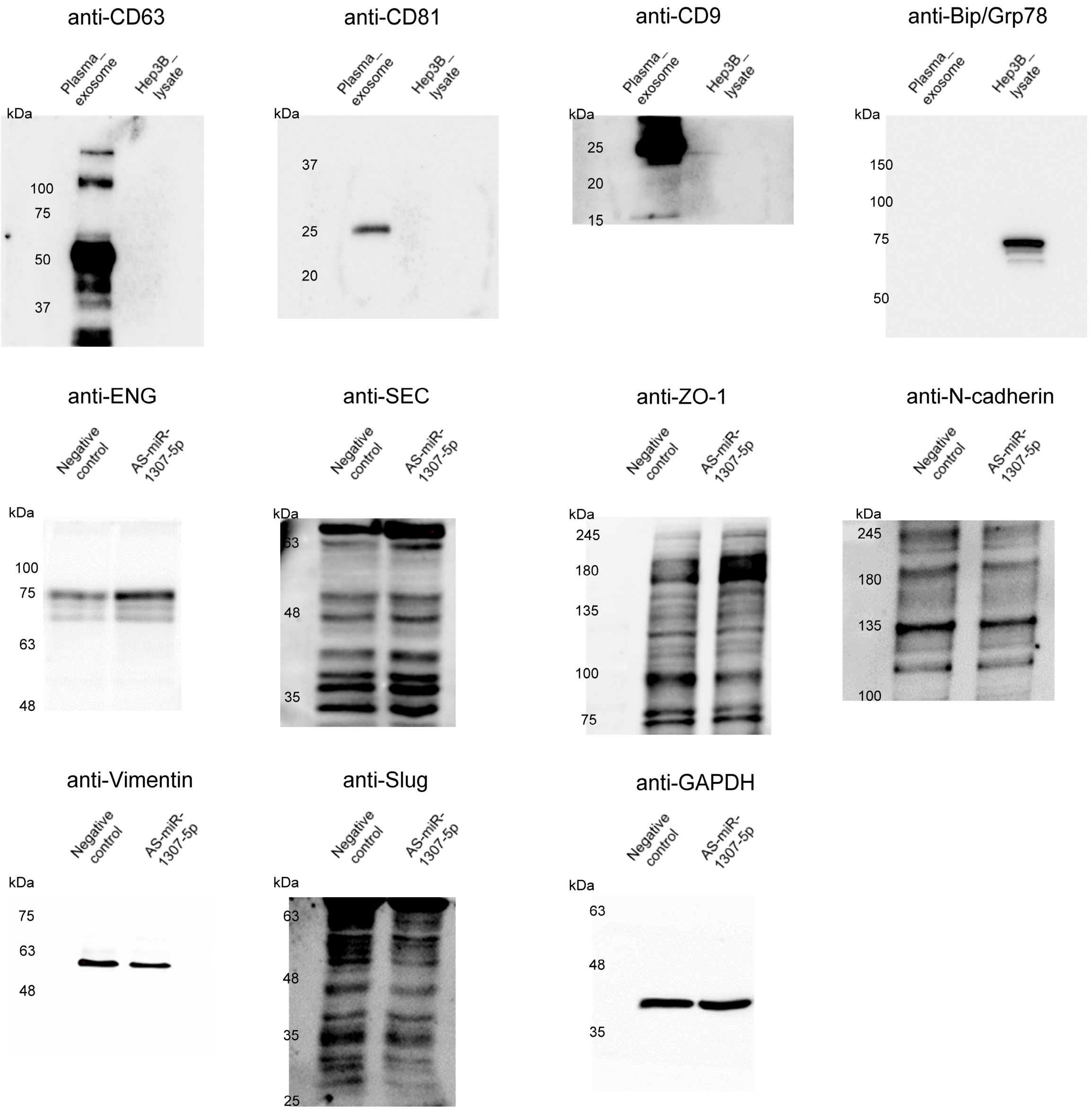

Figure S1. The whole blot (uncropped blots) showing all the bands with all molecular weight markers,.

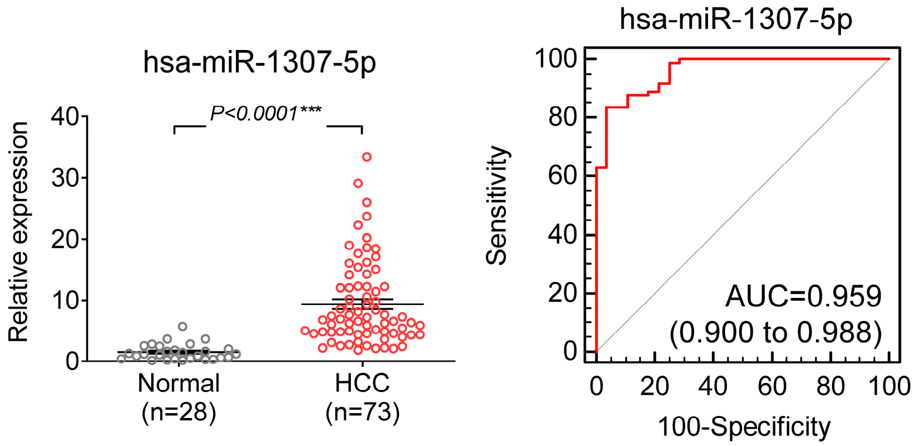

Figure S2. Serum exo-miR-1307-5p expression and ROC curves in normal healthy controls and the patients with HCC.

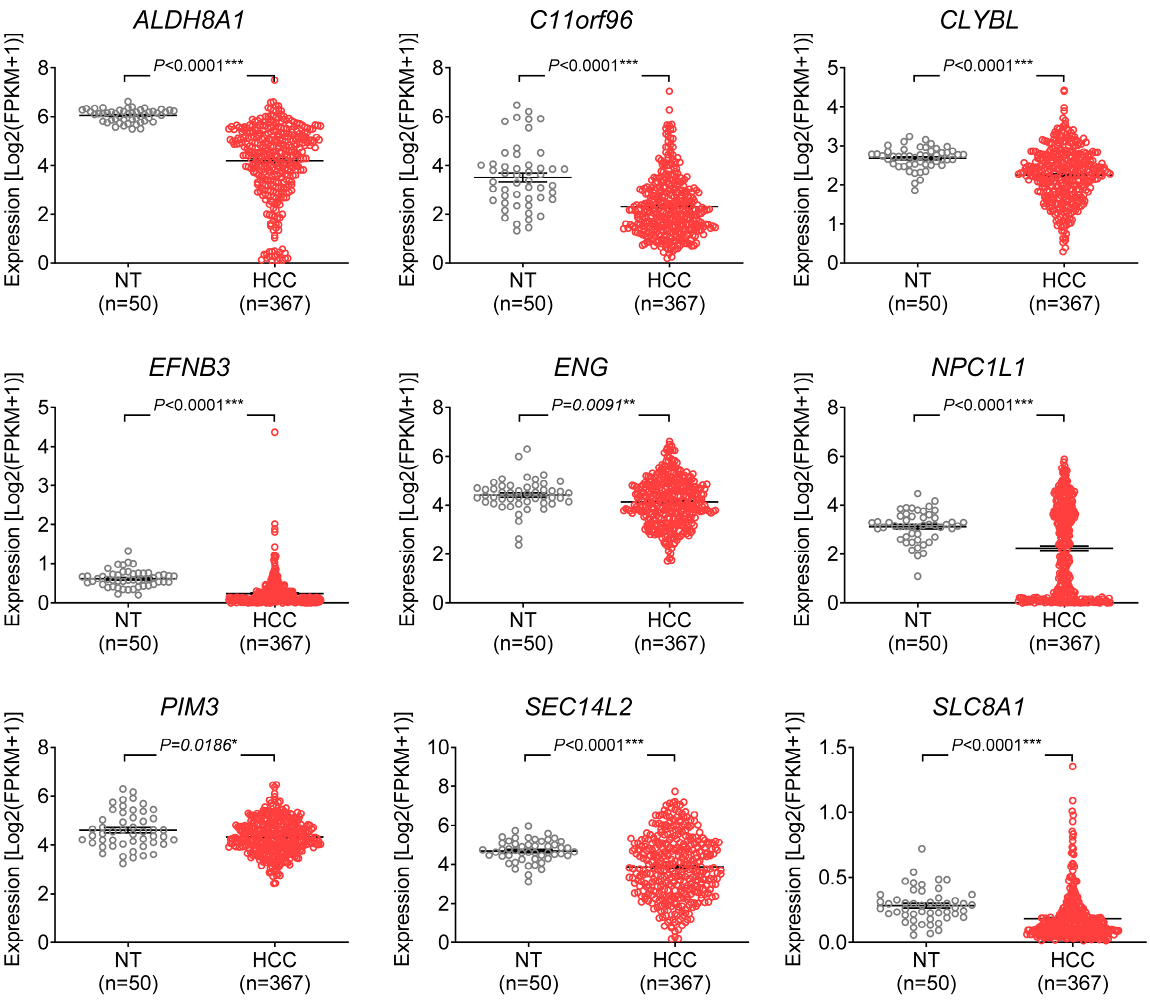

Figure S3. Expression of the nine target gene candidates of miR-1307-5p in The Cancer Genomic Atlas Liver Hepatocellular Carcinoma (TCGA_LIHC).

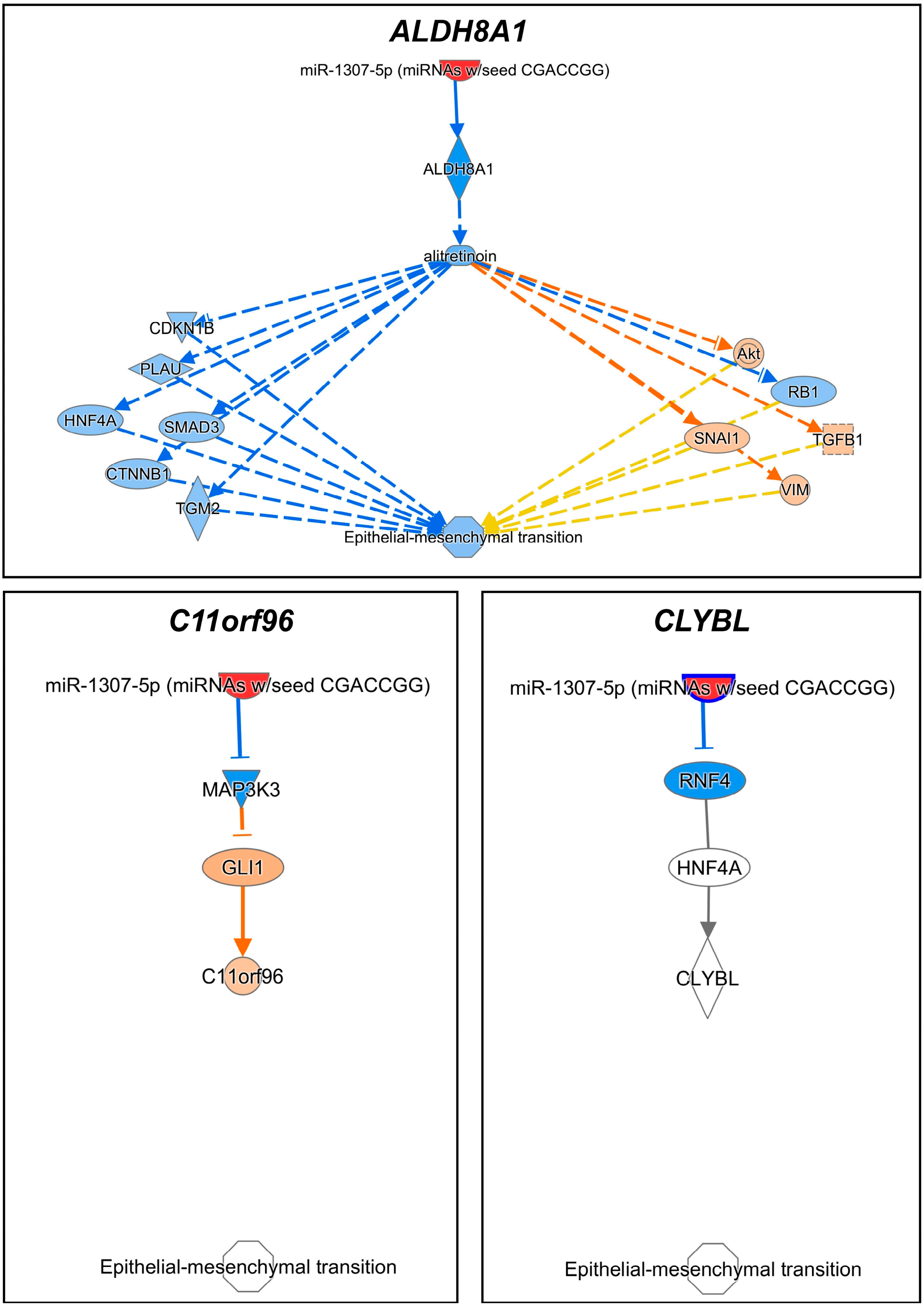

Figure S4. Pathway analysis with a functional annotation of the epithelial–mesenchymal transition (EMT) using the Ingenuity Pathway Analysis (IPA) software on miR-1307-5p and the target gene candidates, *ALDH8A1*, *C11orf96*, and *CLYBL*.

Table 1. miRNA sequence used for qRT-PCR.

Gene	Accession No.	Mature sequence	Primer sequence
hsa-miR-106b-5p	MIMAT0000680	5′-UAAAGUGCUGACAGUGCAGAU –3′	5'-TAAAGTGCTGACAGTGCAGAT –3'
hsa-miR-1307-5p	MIMAT0022727	5′-UCGACCGGACCUCGACCGGCU-3′	5'-TCGACCGGACCTCGACCGGCT -3'
hsa-miR-340-5p	MIMAT0004692	5′-UUAUAAAGCAAUGAGACUGAUU-3′	5'-TTATAAAGCAATGAGACTGATT-3'
hsa-miR-1228-3p	MIMAT0005583	5′-UCACACCUGCCUCGCCCCCC -3′	5'-TCACACCTGCCTCGCCCCCC-3'
